# The main mediating lipid species in cholesterol-induced colorectal cancer risk

**DOI:** 10.3389/fnut.2025.1453523

**Published:** 2025-05-16

**Authors:** Yuanyuan Wang, Tiantian Wang, Xinru Shen, Zhitu Zhu

**Affiliations:** ^1^Cancer Clinical Research Ward, The First Affiliated Hospital of Jinzhou Medical University, Jinzhou, China; ^2^Department of Oncology, The First Affiliated Hospital of Jinzhou Medical University, Jinzhou, China; ^3^Liaoning Provincial Key Laboratory of Clinical Oncology Metabonomics, Institute of Clinical Bioinformatics, Cancer Center of Jinzhou Medical University, The First Affiliated Hospital of Jinzhou Medical University, Jinzhou, China

**Keywords:** lipids, colorectal cancer, Mendelian randomization, cancer risk, cholesterol

## Abstract

**Background:**

Research has indicated that both total cholesterol (TC) and low-density lipoprotein (LDL) cholesterol levels may impact the risk of colorectal cancer (CRC). However, as TC and LDL cholesterol consist of multiple lipid species, it remains uncertain which specific species contribute to this risk. Therefore, this study plans to search for the major lipid species that influence the risk of CRC.

**Methods:**

Initially, a two-sample Mendelian randomization analyses was employed to examine the association between 179 lipid levels and the risk of CRC. Subsequent to this, a meta-analysis was conducted on the results of Mendelian randomization analyses in four CRC cohorts to further determine the relationship between the implicated lipids and CRC risk. Reverse Mendelian randomization was utilized to investigate the potential reverse causal relationship between the relevant lipids and CRC. Lastly, a two-step Mendelian randomization analysis was employed to assess whether the associated lipids acted as mediators in the relationship between TC and LDL cholesterol levels and CRC risk.

**Results:**

Our study identified five lipid levels across multiple cohorts that were significantly associated with the risk of CRC. Meta-analysis results indicated a positive correlation between sterol ester (27:1/14:0) and sterol ester (27:1/16:0) levels and CRC risk (*p* < 0.05), with no evidence of reverse causality. Furthermore, sterol ester (27:1/14:0) and sterol ester (27:1/16:0) were found to mediate the relationship between TC and LDL cholesterol levels and the risk of CRC. Specifically, sterol ester (27:1/14:0) accounted for 87.9 and 93.3% of the effects of TC and LDL cholesterol on CRC risk, while sterol ester (27:1/16:0) mediated 44.3 and 44.6% of these effects, respectively.

**Conclusion:**

Sterol esters (27:1/14:0) and (27:1/16:0) are significant lipids that influence the risk of CRC and act as mediators of TC and LDL cholesterol increasing the risk of CRC.

## 1 Introduction

Colorectal cancer (CRC) is the most common digestive system cancer, ranking as the third most prevalent cancer globally and the second leading cause of cancer-related mortality (1). In 2022, an estimated 153,020 new cases of CRC and 52,550 deaths were reported worldwide (2). Identifying risk factors, prevention, and early interventions are crucial due to the high incidence and mortality of CRC.

Research has indicated a correlation between blood lipids and various cancer risks, particularly in patients with hyperlipidemia who exhibit an elevated risk of colon, prostate, and testicular cancer (3). Alterations in total cholesterol (TC) and low-density lipoprotein (LDL) cholesterol have been shown to increase the risk of CRC (4, 5). Nevertheless, it is important to note that both TC and LDL cholesterol consist of cholesterol as well as various other lipids (6, 7). At present, the specific lipid species that influence the risk of CRC remains unidentified.

Mendelian randomization (MR) has become a prominent method in investigating disease etiology, particularly in the absence of randomized controlled trials (8). By utilizing single nucleotide polymorphisms (SNPs) as instrumental variables (IV), MR allows for the assessment of causal relationships between exposures and outcomes (9). The IV model effectively mitigates confounding in observational studies by assuming random assignment of genotypes during gamete formation, thereby addressing bias stemming from unmeasured confounders on causal inference (10).

Benefit from the progress of modern efficient lipidomics technologies, Ottensmann et al. have acquired genome-wide association study (GWAS) data on 179 plasma lipid species, including 16 sterols (ST), 15 sphingolipids (SL), 44 glycerolipids (GL), and 104 glycerophospholipids (GP) (6).

The current research conducted an analysis on the correlation between 179 plasma lipid species and the risk of CRC using MR analysis. The findings suggest a significant positive association between sterol ester (27:1/14:0) and sterol ester (27:1/16:0) levels and the risk of CRC. As constituents of TC and LDL, these lipid components act as mediators in the relationship between TC and LDL levels and the risk of CRC. Given the crucial role of lipids in human physiological processes, targeted regulation of specific lipid species, rather than a broad approach targeting all lipids, at the dietary, nutritional, or therapeutic levels may represent a viable strategy for the prevention of CRC.

## 2 Results

### 2.1 Association of levels of 179 plasma lipids with risk of CRC

**Figure 1:**
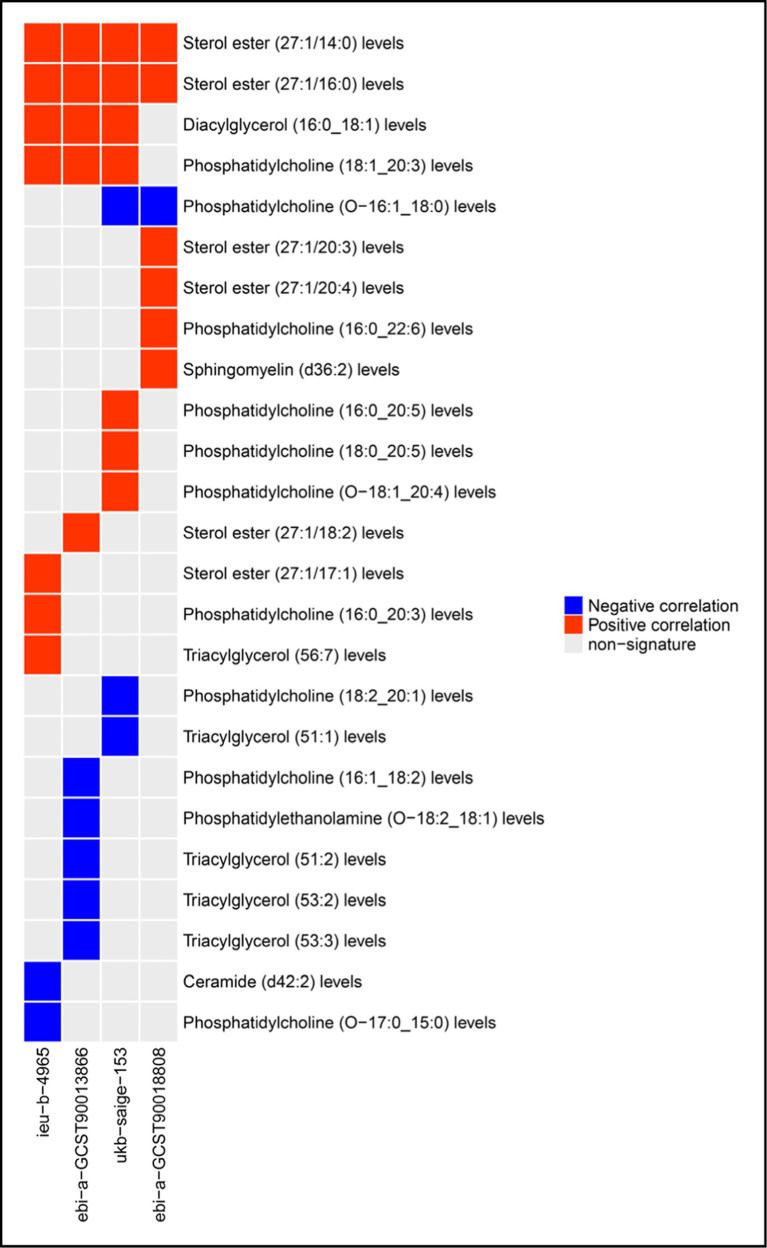
Plasma lipid species associated with CRC risk.

Two-sample Mendelian randomization was utilized to examine the correlation between 179 plasma lipid levels and the risk of CRC. Results indicated that 25 lipid species were significantly linked to CRC risk in one or more cohorts. Specifically, phosphatidylcholine (O-16:1_18:0) levels demonstrated an inverse relationship with CRC risk in two cohorts, while phosphatidylcholine (18:1_20:3) levels and diacylglycerol (16:0_18:1) levels exhibited a positive association with CRC risk in three cohorts. Additionally, sterol ester (27:1/14:0) levels and sterol ester (27:1/16:0) levels were found to be positively correlated with CRC risk in all cohorts analyzed. Furthermore, a total of 21 lipids were identified as being linked to the risk of CRC in a single cohort. These findings are presented in Figure 1. The MR analysis results and tests for heterogeneity and pleiotropy for statistically significant lipid and CRC risk in each cohort are in Supplementary Tables 1–5. The outcomes of the corresponding leave-one-out sensitivity analysis examining the impact of plasma lipid species on CRC, in addition to the funnel plots, scatter plots, and forest plots associated with the primary MR analysis, are presented in Supplementary Figures 1–4.

### 2.2 Meta-analysis of the relationship between lipids and risk of CRC

**Figure 2:**
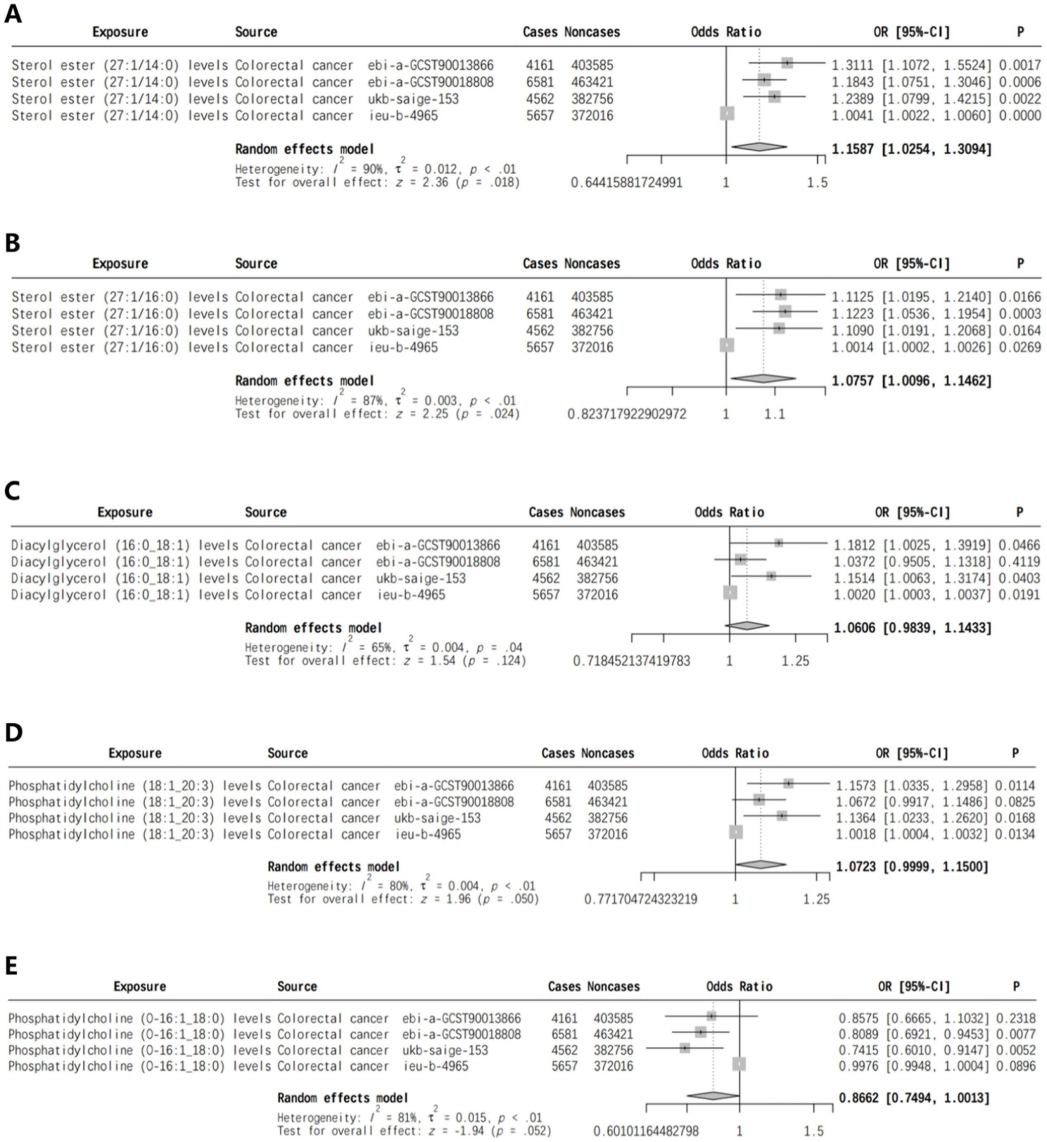
Meta-analysis of lipids associated with CRC risk in two or more cohorts [**(A)** Sterol ester (27:1/14:0) levels, **(B)** Sterol ester (27:1/16:0) levels, **(C)** Diacylglycerol (16:0_18:1) levels, **(D)** Phosphatidylcholine (18:1_20:3) levels, **(E)** Phosphatidylcholine (O-16:1_18:0) levels].

We identified lipids linked to CRC risk in multiple cohorts and conducted a meta-analysis on the results of the IVW analysis. Sterol ester (27:1/14:0) and Sterol ester (27:1/16:0) levels were found to be positively associated with CRC risk, with *p*-values of 0.018 and 0.024, respectively (Figures 2A,B). Meta-analysis found no significant association between Diacyl glycerol (16:0 _ 18:1) levels and CRC risk (*p* = 0.124) (Figure 2C). Phosphatidylcholine (18:1 _ 20:3) levels were positively linked to CRC risk (*p* = 0.050) (Figure 2D), while Phosphatidylcholine (O-16: 1 _ 18:0) levels were inversely associated with risk (*p* = 0.052) (Figure 2E).

### 2.3 Reverse Mendelian randomization of sterol ester (27:1/14:0) levels, sterol ester (27:1/16:0) levels, and risk of CRC

The reverse Mendelian randomization analysis of sterol ester (27:1/14:0) levels and sterol ester (27:1/16:0) levels in relation to CRC across four cohorts revealed a lack of significant correlation between CRC and sterol ester (27:1/14:0) levels and sterol ester (27:1/16:0) levels (Table 1).

**Table 1:** Reverse Mendelian randomization of sterol ester (27:1/14:0) levels, sterol ester (27:1/16:0) levels and risk of CRC.

id.exposure	Exposure	Outcome	Method	No. SNPs	OR (95%CI)	*p* value
ebi-a-GCST90013866	Colorectal cancer	Sterol ester (27:1/14:0) levels	IVW	2	0.961(0.808–1.144)	0.657
ebi-a-GCST90018808	Colorectal cancer	Sterol ester (27:1/14:0) levels	IVW	14	0.986(0.899–1.082)	0.763
ieu-b-4965	Colorectal cancer	Sterol ester (27:1/14:0) levels	IVW	5	0.160(0.000–153.361)	0.601
ukb-saige-153	Colorectal cancer	Sterol ester (27:1/14:0) levels	IVW	3	0.950(0.845–1.069)	0.395
ebi-a-GCST90013866	Colorectal cancer	Sterol ester (27:1/16:0) levels	IVW	3	0.981(0.836–1.151)	0.814
ebi-a-GCST90018808	Colorectal cancer	Sterol ester (27:1/16:0) levels	IVW	11	0.971(0.875–1.076)	0.572
ieu-b-4965	Colorectal cancer	Sterol ester (27:1/16:0) levels	IVW	3	0.084(0.000–620.407)	0.585
ukb-saige-153	Colorectal cancer	Sterol ester (27:1/16:0) levels	IVW	2	0.973(0.844–1.123)	0.713

### 2.4 Mediation analysis

Our study revealed that elevated levels of TC and LDL cholesterol were associated with an increased risk of CRC (Table 2). Subsequently, we employed a two-step Mendelian randomization approach to investigate the mediating effects of sterol ester (27:1/14:0) and sterol ester (27:1/16:0) on the relationship between TC, LDL cholesterol, and CRC risk. Our findings indicated that sterol ester (27:1/14:0) mediated 87.9 and 93.3% of the effects of TC and LDL cholesterol on CRC risk, respectively. Additionally, sterol ester (27:1/16:0) was found to mediate 44.3 and 44.6% of the effects of TC and LDL cholesterol on CRC risk, respectively. The *p* values of Interactive Mediation Tests were all less than 0.05. The results are shown in Table 3. Relevant MR analysis results and heterogeneity and pleiotropy test results can be found in Supplementary materials 1–4.

**Table 2:** The relationship between TC, LDL, HDL levels, and the risk of CRC.

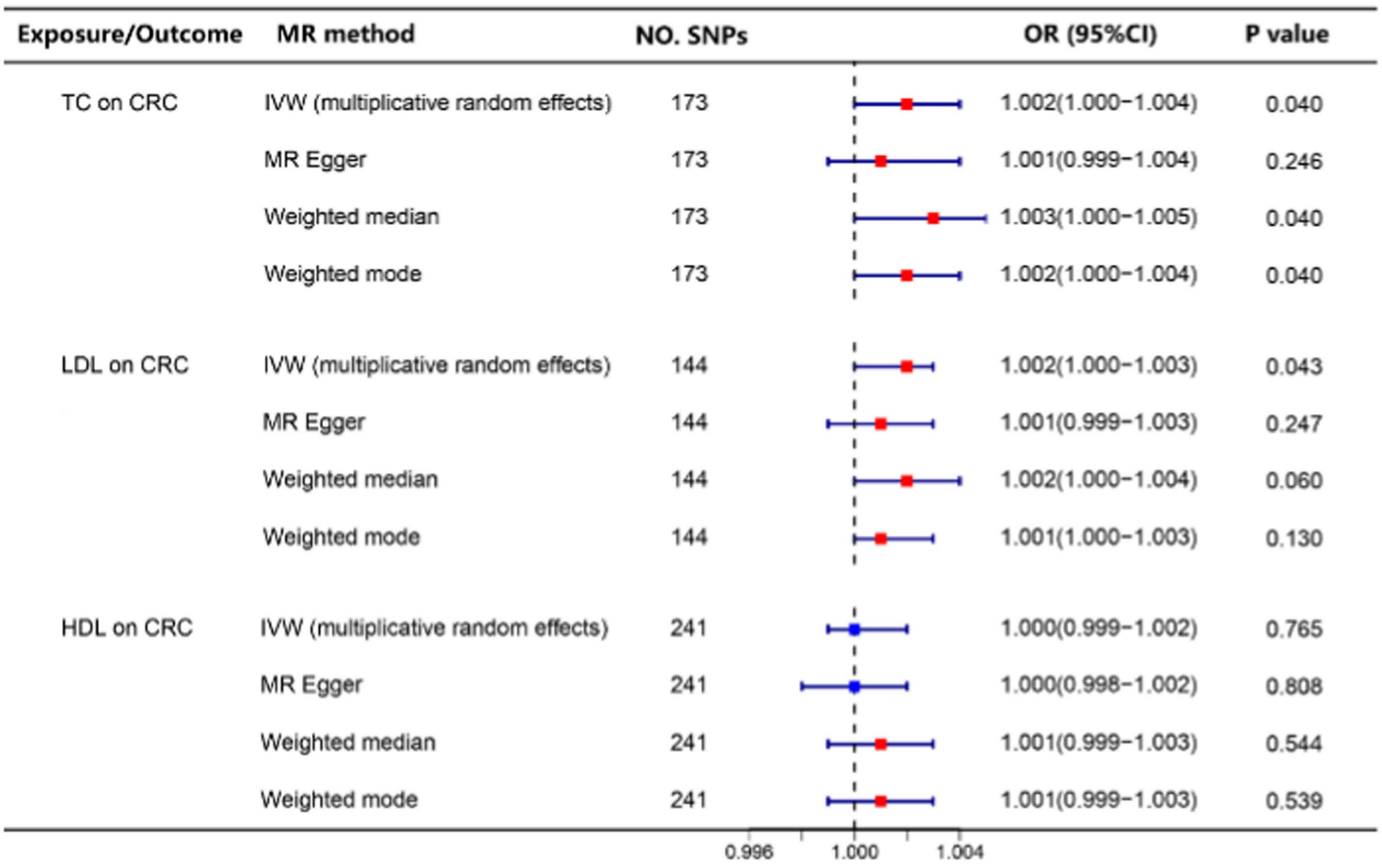

**Table 3:** Proportion of the association between TC or LDL and CRC mediated by sterol ester (27:1/14:0) and sterol ester (27:1/16:0).

Mediators	Exposure	Outcome	Mediation effect in total effect	95%CI	*p* value of Interactive Mediation Tests
Sterol ester (27:1/14:0)	Total cholesterol	Colorectal cancer	87.9%	41.8~134.0%	0.000
	LDL cholesterol	Colorectal cancer	93.3%	44.6~141.9%	0.000
Sterol ester (27:1/16:0)	Total cholesterol	Colorectal cancer	44.3%	3.3~85.4%	0.033
	LDL cholesterol	Colorectal cancer	44.6%	2.5~86.7%	0.036

## 3 Discussion

Dyslipidemia has been found to be significantly associated with various types of cancers. A study focusing on the relationship between dyslipidemia and breast cancer revealed a notably higher prevalence of dyslipidemia among breast cancer patients compared to both healthy individuals and those with benign breast disease. Specifically, levels of TC, triglycerides, and LDL cholesterol were markedly elevated in the breast cancer cohort in comparison to individuals with benign breast disease and healthy controls, with the exception of high-density lipoprotein (HDL) cholesterol (11). Furthermore, high levels of HDL-C and apolipoproteins A1 (apoA1) have been shown to potentially decrease the risk of developing breast cancer (12).

Research on hyperlipidemia and its association with bladder cancer has revealed that individuals with this condition, particularly young adult men, are at an increased risk for developing bladder cancer (13). Furthermore, patients with hyperlipidemia are also more susceptible to colon, prostate, and testicular cancer (3). Additionally, elevated levels of cholesterol have been linked to a higher risk of ovarian cancer, while high levels of HDL-C have shown a protective effect against this type of cancer (14). Studies have also demonstrated that TC and LDL levels are correlated with an elevated risk of CRC (4, 5), whereas higher levels of serum HDL are associated with a decreased risk of colon cancer (15).

TC is composed of free cholesterol and various cholesterol esters (6). Within lipoproteins, VLDL contains 12–15% cholesteryl esters and 8–10% cholesterol, while IDL contains 32–35% cholesteryl esters and 8–10% cholesterol. LDL, smaller than IDL, harbors 37–48% cholesteryl esters and 8–10% cholesterol. HDL contains 15–30% cholesteryl esters and 2–10% cholesterol (7). The specific lipid species that may impact CRC risk remain unclear.

This study examined the correlation between 179 plasma lipid species and the likelihood of developing CRC, encompassing 16 sterols (ST), 15 sphingolipids (SL), 44 glycerolipids (GL), and 104 glycerophospholipids (GP) (6).

To ensure the accuracy of the results, we predominantly utilized MR analysis as our methodological approach. MR is a sophisticated technique that leverages genetic variants as instrumental variables to deduce causal relationships, primarily employed to examine the associations between genetic variations and diseases. The pertinent data are chiefly concerned with the presence or absence of diseases. In comparison to traditional observational studies, MR offers substantial advantages by effectively mitigating biases arising from confounding factors and reverse causation, thereby facilitating more accurate causal inferences (16, 17). These confounding factors include variables such as gender, age, and environmental influences, which are often challenging to fully control in observational studies (18). Furthermore, MR enhances the reliability of causal inference through various methodologies. For example, the implementation of two-sample MR, bidirectional MR, and network MR enables researchers to confirm the robustness of causal relationships across diverse research contexts and to investigate potential pleiotropic pathways (17, 19). MR is a powerful tool for causal inference, effectively evaluating a risk factor’s impact on disease outcomes. It surpasses traditional observational studies’ limitations and offers crucial insights into the causal mechanisms of complex diseases.

In this study, bidirectional MR analysis was primarily utilized to mitigate the potential for reverse causation, thereby ensuring the validity of the results. Additionally, to enhance the robustness of the findings, we employed GWAS data on colorectal cancer from four European cohorts, identifying 25 lipid species potentially associated with colorectal cancer risk. To further ensure the precision and reliability of the results, a meta-analysis was conducted on the MR analysis outcomes from the four datasets.

Ultimately, our study identified a positive association between the levels of two cholesterol esters, sterol ester (27:1/14:0) and sterol ester (27:1/16:0), and the risk of CRC across all cohorts. The meta-analysis results also indicated statistical significance with *p* values less than 0.05.

Subsequent analysis revealed that sterol ester (27:1/14:0) and sterol ester (27:1/16:0), act as mediating factors in the relationship between TC and LDL levels and the risk of CRC. Given the significant physiological roles of lipids in human biology, focusing on specific lipids rather than all lipids in areas such as diet, nutrition, and therapeutic interventions may be a better strategy.

Limitations and deficiencies: This study found two lipid species linked to CRC risk, but did not have enough evidence to draw conclusions about other lipid components. For instance, certain lipid have been linked to CRC risk in one cohort but not in others. Further research is needed to understand their specific relationship to the disease. Some lipid species were suggested to be associated with CRC risk in multiple cohorts, but the meta-analysis did not show statistical significance, particularly for Phosphatidylcholine (O-16:1_18:0) and Phosphatidylcholine (18:1_20:3) with a *p* value of 0.052 and 0.050. More investigation is needed to determine their relationship with CRC risk.

## 4 Methods

### 4.1 Study design

This study initially examined the correlation between 179 lipid species and the risk of CRC across multiple cohorts using a two-sample Mendelian randomization (MR) approach. Subsequently, a meta-analysis was conducted to further clarify the association between lipids and CRC risk, while also investigating the possibility of reverse causation through reverse MR. Finally, a two-step Mendelian randomization method was employed to assess whether the identified lipids acted as mediators in the relationship between TC and LDL cholesterol levels and the risk of CRC.

### 4.2 Data sources

The Genome-wide association studies (GWAS) data for 179 lipid species in the Finnish population were sourced from Ottensmann et al. (6), while data on TC, LDL, and HDL levels were obtained from Sakaue et al. (20). Additionally, nearly 5 years of GWAS data on CRC were downloaded from the IEU database^1^ (21), with the exclusion of data from the FinnGen dataset to prevent population duplication. Further GWAS data on CRC from Taliun D et al. (22) were also utilized, all of which pertained to European populations. Specific details regarding the data can be found in Table 4. Because ebi-a-gcst90013862 and ebi-a-gcst90013866 are resulting from distinct correction methods applied to the same population, only the cohort ebi-a-GCST90013866 was chosen for analysis. Details of all data including 179 lipid species, cholesterol, and colorectal cancer can be found in Supplementary materials 5–7.

**Table 4:** Summary of the data sets.

Trait	ID	Population	Sample size	Year	Author
Total cholesterol	ebi-a-GCST90018974	European	344,278	2021	Sakaue S
HDL cholesterol	ebi-a-GCST90018956	European	315,133	2021	Sakaue S
LDL cholesterol	ebi-a-GCST90018961	European	343,621	2021	Sakaue S
Colorectal cancer	ebi-a-GCST90018808	European	470,002	2021	Sakaue S
	ieu-b-4965	European	377,673	2021	Burrows
	ebi-a-GCST90013862	European	407,746	2021	Mbatchou J
	ebi-a-GCST90013866	European	407,746	2021	Mbatchou J
	ukb-saige-153	European	387,318	2021	Taliun D

### 4.3 Selection of IV

In the Mendelian randomization (MR) analysis conducted in this study, SNPs that met the genome-wide significance threshold (*p* < 5 × 10^–6^) and exhibited no linkage disequilibrium (LD) with other SNPs (r^2^ < 0.001 within a 10,000 kb clumping window) were utilized as IV for the exposures. In the reverse MR analysis, SNPs meeting the genome-wide significance threshold (*p* < 5 × 10^–8^) and displaying no LD with other SNPs (r^2^ < 0.001 within a 10,000 kb clumping window) were employed as IV for the exposures. Outliers that may influence causal effects in the MR-PRESSO global test were identified and eliminated (23), along with SNPs associated with the outcome at a significance level of *p* < 5 × 10^–5^. Furthermore, the Steiger test method was employed to identify SNPs showing stronger associations with the outcome variable relative to the exposure variable, and these SNPs were also excluded from the analysis.

### 4.4 Sensitivity analysis

The study utilized the χ^2^ Q test to evaluate the diversity in causal impacts of various variants, where a significance level below 0.05 denoted heterogeneity. Additionally, the MR-Egger intercept analysis was employed to investigate horizontal pleiotropy, a condition in which IV affect both the exposure and outcome through a non-causal route. The findings revealed no substantial indication of horizontal pleiotropy (MR-Egger intercept <0.01, *p*-value >0.05).

### 4.5 Mediation analysis

**Figure 3:**
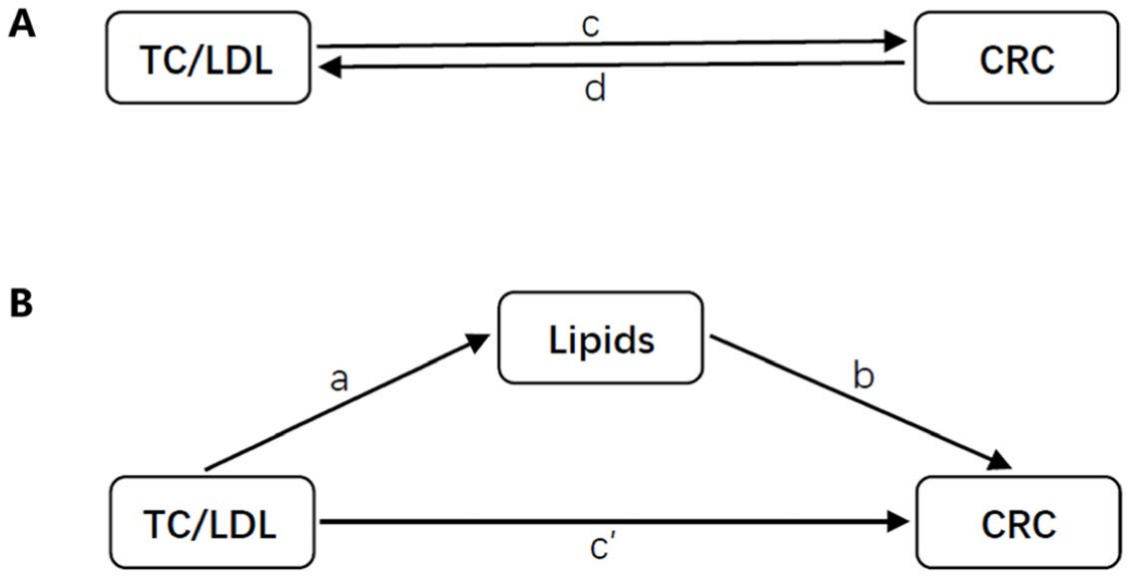
Diagrams illustrating associations examined in this study. **(A)** The total effect between TC or LDL and CRC. The total effect is represented by c when genetically predicted TC or LDL is the exposure and CRC is the outcome, and by d when genetically predicted CRC is the exposure and TC or LDL is the outcome. **(B)** The total effect was further analyzed by decomposing it into indirect effects using a two-step approach (where a represents the total effect of TC or LDL on CRC, and b represents the effect of CRC on TC or LDL) and the product method (a × b), as well as direct effects (c′ = c – a × b). The proportion mediated was calculated by dividing the indirect effect by the total effect.

In order to assess the extent to which relevant lipids mediate the relationship between TC or LDL and the risk of CRC, a two-step Mendelian randomization (MR) method was employed. The total effect was partitioned into an indirect effect (mediated by lipids) and a direct effect (not mediated by lipids) (24). The proportion of mediation was determined by dividing the indirect effect by the total effect (Figure 3).

### 4.6 Statistical analysis

We utilized R (version 4.3.0) along with the packages “TwoSampleMR,” “MendelR,” and “MRPRESSO” for conducting all Mendelian randomization (MR) and meta-analyses. The inverse variance weighted (IVW) method served as our primary MR approach (25), complemented by additional methods such as MR Egger, Weighted Median, and Weighted Mode. The IVW method was employed in the absence of heterogeneity, while the IVW (multiplicative random effects) method was utilized in cases of heterogeneity. In instances of pleiotropic effects, preference was given to the MR Egger method over IVW. A random effects model was used for the meta-analysis due to data heterogeneity. A significance level of *p* < 0.05 was considered statistically significant for all analyses.

## Data Availability

The original contributions presented in the study are included in the article/Supplementary material, further inquiries can be directed to the corresponding author.

## Footnotes

^1^ https://gwas.mrcieu.ac.uk/
