# Supplementary material for: The main mediating lipid species in cholesterol-induced colorectal cancer risk
Source: Front Nutr. 2025 May 16;12:1453523. doi: 10.3389/fnut.2025.1453523 (PMC12124121; doi:10.3389/fnut.2025.1453523)
Supplement: Supplementary file 1 [file Table_1.docx]

| **Outcome** **(ebi-a-GCST90013866)** | | | | | | |
| --- | --- | --- | --- | --- | --- | --- |
| **Exposure** | **Method** | **NSNP** | **OR（95%CI）** | **P value** | **P value. Heterogeneity** | **P value. Pleiotropy** |
| Sterol ester (27:1/14:0) levels | IVW | 10 | 1.311 ( 1.107 - 1.552 ) | 0.002 | 0.944 | 0.898 |
|  | MR Egger | 10 | 1.276 ( 0.828 - 1.967 ) | 0.301 | 0.904 |  |
|  | Weighted median | 10 | 1.271 ( 1.011 - 1.599 ) | 0.040 |  |  |
|  | Weighted mode | 10 | 1.150 ( 0.835 - 1.583 ) | 0.414 |  |  |
| Sterol ester (27:1/16:0) levels | IVW | 23 | 1.113 ( 1.020 - 1.214 ) | 0.017 | 0.567 | 0.669 |
|  | MR Egger | 23 | 1.149 ( 0.971 - 1.359 ) | 0.122 | 0.517 |  |
|  | Weighted median | 23 | 1.160 ( 1.019 - 1.319 ) | 0.024 |  |  |
|  | Weighted mode | 23 | 1.166 ( 1.015 - 1.338 ) | 0.040 |  |  |
| Sterol ester (27:1/18:2) levels | IVW | 16 | 1.113 ( 1.005 - 1.233 ) | 0.040 | 0.375 | 0.895 |
|  | MR Egger | 16 | 1.102 ( 0.925 - 1.314 ) | 0.295 | 0.308 |  |
|  | Weighted median | 16 | 1.141 ( 0.990 - 1.315 ) | 0.069 |  |  |
|  | Weighted mode | 16 | 1.135 ( 0.971 - 1.327 ) | 0.133 |  |  |
| Diacylglycerol (16:0_18:1) levels | IVW | 7 | 1.181 ( 1.002 - 1.392 ) | 0.047 | 0.384 | 0.274 |
|  | MR Egger | 7 | 1.374 ( 1.029 - 1.835 ) | 0.084 | 0.434 |  |
|  | Weighted median | 7 | 1.232 ( 0.995 - 1.526 ) | 0.056 |  |  |
|  | Weighted mode | 7 | 1.291 ( 1.007 - 1.654 ) | 0.090 |  |  |
| Phosphatidylcholine (16:1_18:2) levels | IVW | 15 | 0.905 ( 0.827 - 0.991 ) | 0.031 | 0.629 | 0.830 |
|  | MR Egger | 15 | 0.890 ( 0.746 - 1.061 ) | 0.217 | 0.555 |  |
|  | Weighted median | 15 | 0.891 ( 0.795 - 0.998 ) | 0.046 |  |  |
|  | Weighted mode | 15 | 0.893 ( 0.797 - 1.001 ) | 0.072 |  |  |
| Phosphatidylcholine (18:1_20:3) levels | IVW | 15 | 1.157 ( 1.034 - 1.296 ) | 0.011 | 0.446 | 0.374 |
|  | MR Egger | 15 | 1.032 ( 0.787 - 1.351 ) | 0.825 | 0.434 |  |
|  | Weighted median | 15 | 1.126 ( 0.952 - 1.332 ) | 0.165 |  |  |
|  | Weighted mode | 15 | 1.119 ( 0.880 - 1.423 ) | 0.376 |  |  |
| Phosphatidylethanolamine (O-18:2_18:1) levels | IVW | 12 | 0.881 ( 0.778 - 0.998 ) | 0.046 | 0.663 | 0.449 |
|  | MR Egger | 12 | 0.816 ( 0.651 - 1.024 ) | 0.109 | 0.636 |  |
|  | Weighted median | 12 | 0.834 ( 0.702 - 0.991 ) | 0.039 |  |  |
|  | Weighted mode | 12 | 0.803 ( 0.642 - 1.004 ) | 0.081 |  |  |
| Triacylglycerol (51:2) levels | IVW | 9 | 0.845 ( 0.719 - 0.993 ) | 0.041 | 0.995 | 0.849 |
|  | MR Egger | 9 | 0.869 ( 0.631 - 1.198 ) | 0.420 | 0.987 |  |
|  | Weighted median | 9 | 0.829 ( 0.678 - 1.014 ) | 0.068 |  |  |
|  | Weighted mode | 9 | 0.837 ( 0.654 - 1.072 ) | 0.196 |  |  |
| Triacylglycerol (53:2) levels | IVW | 13 | 0.826 ( 0.719 - 0.949 ) | 0.007 | 0.999 | 0.999 |
|  | MR Egger | 13 | 0.826 ( 0.450 - 1.516 ) | 0.550 | 0.998 |  |
|  | Weighted median | 13 | 0.817 ( 0.680 - 0.982 ) | 0.032 |  |  |
|  | Weighted mode | 13 | 0.833 ( 0.627 - 1.106 ) | 0.231 |  |  |
| Triacylglycerol (53:3) levels | IVW | 18 | 0.889 ( 0.793 - 0.998 ) | 0.046 | 0.962 | 0.637 |
|  | MR Egger | 18 | 0.961 ( 0.686 - 1.346 ) | 0.821 | 0.950 |  |
|  | Weighted median | 18 | 0.878 ( 0.751 - 1.027 ) | 0.104 |  |  |
|  | Weighted mode | 18 | 0.880 ( 0.691 - 1.121 ) | 0.316 |  |  |

**Supplementary Table 1. MR analysis results of plasma lipid levels and colorectal cancer risk (ebi-a-GCST90013866)**

| **Outcome** **(ebi-a-GCST90018808)** | | | | | | |
| --- | --- | --- | --- | --- | --- | --- |
| **Exposure** | **Method** | **NSNP** | **OR（95%CI）** | **P value** | **P value. Heterogeneity** | **P value. Pleiotrop** |
| Sterol ester (27:1/14:0) levels | IVW | 13 | 1.184 ( 1.075 - 1.305 ) | 0.001 | 0.985 | 0.941 |
|  | MR Egger | 13 | 1.195 ( 0.925 - 1.545 ) | 0.200 | 0.973 |  |
|  | Weighted median | 13 | 1.159 ( 1.023 - 1.313 ) | 0.020 |  |  |
|  | Weighted mode | 13 | 1.133 ( 0.938 - 1.368 ) | 0.220 |  |  |
| Sterol ester (27:1/16:0) levels | IVW | 23 | 1.122 ( 1.054 - 1.195 ) | 0.000 | 0.292 | 0.665 |
|  | MR Egger | 23 | 1.095 ( 0.963 - 1.244 ) | 0.180 | 0.252 |  |
|  | Weighted median | 23 | 1.103 ( 1.005 - 1.209 ) | 0.038 |  |  |
|  | Weighted mode | 23 | 1.088 ( 0.977 - 1.213 ) | 0.139 |  |  |
| Sterol ester (27:1/20:3) levels | IVW | 24 | 1.105 ( 1.042 - 1.172 ) | 0.001 | 0.555 | 0.637 |
|  | MR Egger | 24 | 1.135 ( 1.002 - 1.286 ) | 0.060 | 0.508 |  |
|  | Weighted median | 24 | 1.122 ( 1.020 - 1.233 ) | 0.018 |  |  |
|  | Weighted mode | 24 | 1.129 ( 1.018 - 1.253 ) | 0.031 |  |  |
| Sterol ester (27:1/20:4) levels | IVW | 20 | 1.083 ( 1.019 - 1.150 ) | 0.011 | 0.391 | 0.469 |
|  | MR Egger | 20 | 1.039 ( 0.917 - 1.177 ) | 0.555 | 0.364 |  |
|  | Weighted median | 20 | 1.132 ( 1.038 - 1.235 ) | 0.005 |  |  |
|  | Weighted mode | 20 | 1.107 ( 1.011 - 1.213 ) | 0.041 |  |  |
| Phosphatidylcholine (16:0_22:6) levels | IVW | 13 | 1.136 ( 1.050 - 1.230 ) | 0.002 | 0.158 | 0.604 |
|  | MR Egger | 13 | 1.180 ( 1.005 - 1.386 ) | 0.069 | 0.129 |  |
|  | Weighted median | 13 | 1.097 ( 0.990 - 1.214 ) | 0.076 |  |  |
|  | Weighted mode | 13 | 1.088 ( 0.943 - 1.255 ) | 0.272 |  |  |
| Phosphatidylcholine (O-16:1_18:0) levels | IVW | 7 | 0.809 ( 0.692 - 0.945 ) | 0.008 | 0.097 | 0.411 |
|  | MR Egger | 7 | 0.959 ( 0.640 - 1.436 ) | 0.846 | 0.100 |  |
|  | Weighted median | 7 | 0.806 ( 0.681 - 0.954 ) | 0.012 |  |  |
|  | Weighted mode | 7 | 0.723 ( 0.539 - 0.970 ) | 0.074 |  |  |
| Sphingomyelin (d36:2) levels | IVW | 15 | 1.092 ( 1.007 - 1.183 ) | 0.032 | 0.136 | 0.239 |
|  | MR Egger | 15 | 1.214 ( 1.008 - 1.462 ) | 0.062 | 0.167 |  |
|  | Weighted median | 15 | 1.061 ( 0.953 - 1.182 ) | 0.281 |  |  |
|  | Weighted mode | 15 | 1.048 ( 0.854 - 1.284 ) | 0.662 |  |  |

**Supplementary Table 2. MR analysis results of plasma lipid levels and colorectal cancer risk (ebi-a-GCST90018808)**

| **Outcome (ieu-b-4965)** | | | | | | |
| --- | --- | --- | --- | --- | --- | --- |
| **Exposure** | **Method** | **NSNP** | **OR（95%CI）** | **P value** | **P value. Heterogeneity** | **P value. Pleiotropy** |
| Sterol ester (27:1/14:0) levels | IVW | 13 | 1.004 ( 1.002 - 1.006 ) | 0.000 | 0.655 | 0.810 |
|  | MR Egger | 13 | 1.005 ( 1.000 - 1.009 ) | 0.058 | 0.576 |  |
|  | Weighted median | 13 | 1.004 ( 1.002 - 1.007 ) | 0.001 |  |  |
|  | Weighted mode | 13 | 1.006 ( 1.002 - 1.010 ) | 0.010 |  |  |
| Sterol ester (27:1/16:0) levels | IVW | 24 | 1.001 ( 1.000 - 1.003 ) | 0.027 | 0.205 | 0.670 |
|  | MR Egger | 24 | 1.002 ( 0.999 - 1.004 ) | 0.149 | 0.174 |  |
|  | Weighted median | 24 | 1.002 ( 1.000 - 1.004 ) | 0.012 |  |  |
|  | Weighted mode | 24 | 1.002 ( 1.000 - 1.004 ) | 0.038 |  |  |
| Sterol ester (27:1/17:1) levels | IVW | 8 | 1.003 ( 1.000 - 1.005 ) | 0.038 | 0.410 | 0.514 |
|  | MR Egger | 8 | 1.004 ( 0.999 - 1.009 ) | 0.151 | 0.354 |  |
|  | Weighted median | 8 | 1.002 ( 0.999 - 1.005 ) | 0.238 |  |  |
|  | Weighted mode | 8 | 1.000 ( 0.994 - 1.005 ) | 0.903 |  |  |
| Ceramide (d42:2) levels | IVW | 19 | 0.999 ( 0.997 - 1.000 ) | 0.049 | 0.178 | 0.297 |
|  | MR Egger | 19 | 1.000 ( 0.997 - 1.004 ) | 0.842 | 0.191 |  |
|  | Weighted median | 19 | 0.999 ( 0.997 - 1.001 ) | 0.291 |  |  |
|  | Weighted mode | 19 | 0.999 ( 0.997 - 1.001 ) | 0.525 |  |  |
| Diacylglycerol (16:0_18:1) levels | IVW | 10 | 1.002 ( 1.000 - 1.004 ) | 0.019 | 0.548 | 0.763 |
|  | MR Egger | 10 | 1.002 ( 0.998 - 1.005 ) | 0.362 | 0.456 |  |
|  | Weighted median | 10 | 1.001 ( 0.999 - 1.004 ) | 0.330 |  |  |
|  | Weighted mode | 10 | 1.001 ( 0.998 - 1.004 ) | 0.571 |  |  |
| Phosphatidylcholine (16:0_20:3) levels | IVW | 8 | 1.003 ( 1.001 - 1.004 ) | 0.002 | 0.860 | 0.824 |
|  | MR Egger | 8 | 1.002 ( 0.999 - 1.006 ) | 0.274 | 0.783 |  |
|  | Weighted median | 8 | 1.002 ( 1.000 - 1.004 ) | 0.051 |  |  |
|  | Weighted mode | 8 | 1.002 ( 1.000 - 1.005 ) | 0.097 |  |  |
| Phosphatidylcholine (18:1_20:3) levels | IVW | 16 | 1.002 ( 1.000 - 1.003 ) | 0.013 | 0.486 | 0.464 |
|  | MR Egger | 16 | 1.001 ( 0.998 - 1.004 ) | 0.676 | 0.453 |  |
|  | Weighted median | 16 | 1.002 ( 1.000 - 1.004 ) | 0.104 |  |  |
|  | Weighted mode | 16 | 1.002 ( 0.999 - 1.005 ) | 0.158 |  |  |
| Phosphatidylcholine (O-17:0_15:0) levels | IVW | 10 | 0.998 ( 0.997 - 1.000 ) | 0.036 | 0.882 | 0.824 |
|  | MR Egger | 10 | 0.999 ( 0.996 - 1.001 ) | 0.271 | 0.824 |  |
|  | Weighted median | 10 | 0.998 ( 0.996 - 1.000 ) | 0.067 |  |  |
|  | Weighted mode | 10 | 0.997 ( 0.994 - 1.001 ) | 0.147 |  |  |
| Triacylglycerol (56:7) levels | IVW | 18 | 1.001 ( 1.000 - 1.003 ) | 0.048 | 0.742 | 0.566 |
|  | MR Egger | 18 | 1.003 ( 0.998 - 1.007 ) | 0.256 | 0.704 |  |
|  | Weighted median | 18 | 1.002 ( 1.000 - 1.004 ) | 0.032 |  |  |
|  | Weighted mode | 18 | 1.002 ( 1.000 - 1.005 ) | 0.108 |  |  |

**Supplementary Table 3. MR analysis results of plasma lipid levels and colorectal cancer risk (ieu-b-4965)**

|  | **Colorectal cancer**  **(ukb-saige-153)** | | |  |  |  |
| --- | --- | --- | --- | --- | --- | --- |
| **Exposure** | **Method** | **NSNP** | **OR (95%CI)** | **P value** | **P value. Heterogeneity** | **P value. Pleiotropy** |
| Sterol ester (27:1/14:0) levels | IVW | 14 | 1.239 ( 1.080 - 1.421 ) | 0.002 | 0.695111 | 0.93711 |
|  | MR Egger | 14 | 1.254 ( 0.912 - 1.724 ) | 0.190 | 0.617793 |  |
|  | Weighted median | 14 | 1.160 ( 0.952 - 1.413 ) | 0.142 |  |  |
|  | Weighted mode | 14 | 1.054 ( 0.745 - 1.491 ) | 0.773 |  |  |
| Sterol ester (27:1/16:0) levels | IVW | 24 | 1.109 ( 1.019 - 1.207 ) | 0.016 | 0.470971 | 0.421325 |
|  | MR Egger | 24 | 1.176 ( 0.998 - 1.386 ) | 0.066 | 0.450971 |  |
|  | Weighted median | 24 | 1.170 ( 1.030 - 1.330 ) | 0.016 |  |  |
|  | Weighted mode | 24 | 1.192 ( 1.036 - 1.372 ) | 0.022 |  |  |
| Diacylglycerol (16:0_18:1) levels | IVW | 9 | 1.151 ( 1.006 - 1.317 ) | 0.040 | 0.825938 | 0.294549 |
|  | MR Egger | 9 | 1.305 ( 1.011 - 1.683 ) | 0.080 | 0.880428 |  |
|  | Weighted median | 9 | 1.157 ( 0.972 - 1.376 ) | 0.101 |  |  |
|  | Weighted mode | 9 | 1.114 ( 0.870 - 1.428 ) | 0.417 |  |  |
| Phosphatidylcholine (16:0_20:5) levels | IVW | 12 | 1.135 ( 1.034 - 1.245 ) | 0.008 | 0.970825 | 0.808374 |
|  | MR Egger | 12 | 1.116 ( 0.949 - 1.312 ) | 0.215 | 0.95146 |  |
|  | Weighted median | 12 | 1.131 ( 1.013 - 1.262 ) | 0.028 |  |  |
|  | Weighted mode | 12 | 1.130 ( 1.004 - 1.271 ) | 0.067 |  |  |
| Phosphatidylcholine (18:0_20:5) levels | IVW | 14 | 1.112 ( 1.026 - 1.204 ) | 0.009 | 0.810769 | 0.67422 |
|  | MR Egger | 14 | 1.137 ( 1.000 - 1.292 ) | 0.074 | 0.761476 |  |
|  | Weighted median | 14 | 1.122 ( 1.020 - 1.234 ) | 0.018 |  |  |
|  | Weighted mode | 14 | 1.124 ( 1.018 - 1.240 ) | 0.037 |  |  |
| Phosphatidylcholine (18:1_20:3) levels | IVW | 17 | 1.136 ( 1.023 - 1.262 ) | 0.017 | 0.725473 | 0.416843 |
|  | MR Egger | 17 | 1.037 ( 0.816 - 1.317 ) | 0.771 | 0.711325 |  |
|  | Weighted median | 17 | 1.079 ( 0.936 - 1.245 ) | 0.294 |  |  |
|  | Weighted mode | 17 | 1.086 ( 0.903 - 1.307 ) | 0.392 |  |  |
| Phosphatidylcholine (18:2_20:1) levels | IVW | 10 | 0.881 ( 0.795 - 0.977 ) | 0.016 | 0.939706 | 0.594282 |
|  | MR Egger | 10 | 0.840 ( 0.690 - 1.024 ) | 0.122 | 0.91985 |  |
|  | Weighted median | 10 | 0.901 ( 0.795 - 1.022 ) | 0.104 |  |  |
|  | Weighted mode | 10 | 0.900 ( 0.793 - 1.021 ) | 0.136 |  |  |
| Phosphatidylcholine (O-16:1_18:0) levels | IVW | 7 | 0.741 ( 0.601 - 0.915 ) | 0.005 | 0.163617 | 0.294639 |
|  | MR Egger | 7 | 0.943 ( 0.601 - 1.480 ) | 0.808 | 0.205658 |  |
|  | Weighted median | 7 | 0.852 ( 0.658 - 1.103 ) | 0.225 |  |  |
|  | Weighted mode | 7 | 0.886 ( 0.650 - 1.209 ) | 0.475 |  |  |
| Phosphatidylcholine (O-18:1_20:4) levels | IVW | 9 | 1.171 ( 1.043 - 1.315 ) | 0.007 | 0.424427 | 0.26808 |
|  | MR Egger | 9 | 1.332 ( 1.049 - 1.692 ) | 0.051 | 0.466654 |  |
|  | Weighted median | 9 | 1.195 ( 1.045 - 1.367 ) | 0.009 |  |  |
|  | Weighted mode | 9 | 1.205 ( 1.045 - 1.391 ) | 0.034 |  |  |
| Triacylglycerol (51:1) levels | IVW | 12 | 0.877 ( 0.778 - 0.989 ) | 0.032 | 0.40839 | 0.639815 |
|  | MR Egger | 12 | 0.831 ( 0.647 - 1.068 ) | 0.179 | 0.34482 |  |
|  | Weighted median | 12 | 0.890 ( 0.757 - 1.045 ) | 0.154 |  |  |
|  | Weighted mode | 12 | 0.870 ( 0.701 - 1.081 ) | 0.235 |  |  |

**Supplementary Table 4. MR analysis results of plasma lipid levels and colorectal cancer risk (ukb-saige-153)**

| **Outcome**  **(ebi-a-GCST90013862)** | | | | | | |
| --- | --- | --- | --- | --- | --- | --- |
| **Exposure** | **Method** | **NSNP** | **OR ( 95%CI )** | **P value** | **P value. Heterogeneity** | **P value. Pleiotropy** |
| Sterol ester ( 27:1/14:0 ) levels | IVW | 10 | 1.311 ( 1.107 - 1.552 ) | 0.002 | 0.944 | 0.898 |
|  | MR Egger | 10 | 1.276 ( 0.828 - 1.967 ) | 0.301 | 0.904 |  |
|  | Weighted median | 10 | 1.271 ( 1.023 - 1.579 ) | 0.030 |  |  |
|  | Weighted mode | 10 | 1.150 ( 0.803 - 1.646 ) | 0.465 |  |  |
| Sterol ester ( 27:1/16:0 ) levels | IVW | 23 | 1.114 ( 1.021 - 1.216 ) | 0.015 | 0.552 | 0.660 |
|  | MR Egger | 23 | 1.151 ( 0.973 - 1.362 ) | 0.116 | 0.502 |  |
|  | Weighted median | 23 | 1.160 ( 1.020 - 1.318 ) | 0.023 |  |  |
|  | Weighted mode | 23 | 1.167 ( 1.016 - 1.340 ) | 0.039 |  |  |
| Sterol ester ( 27:1/18:2 ) levels | IVW | 16 | 1.116 ( 1.007 - 1.236 ) | 0.037 | 0.362 | 0.904 |
|  | MR Egger | 16 | 1.106 ( 0.927 - 1.319 ) | 0.283 | 0.296 |  |
|  | Weighted median | 16 | 1.141 ( 0.989 - 1.317 ) | 0.070 |  |  |
|  | Weighted mode | 16 | 1.136 ( 0.970 - 1.329 ) | 0.134 |  |  |
| Diacylglycerol ( 16:0_18:1 ) levels | IVW | 7 | 1.181 ( 1.002 - 1.392 ) | 0.047 | 0.384 | 0.274 |
|  | MR Egger | 7 | 1.374 ( 1.029 - 1.835 ) | 0.084 | 0.434 |  |
|  | Weighted median | 7 | 1.232 ( 0.989 - 1.535 ) | 0.063 |  |  |
|  | Weighted mode | 7 | 1.291 ( 0.993 - 1.677 ) | 0.105 |  |  |
| Phosphatidylcholine ( 16:1_18:2 ) levels | IVW | 15 | 0.905 ( 0.827 - 0.990 ) | 0.030 | 0.628 | 0.834 |
|  | MR Egger | 15 | 0.890 ( 0.746 - 1.061 ) | 0.217 | 0.554 |  |
|  | Weighted median | 15 | 0.891 ( 0.799 - 0.994 ) | 0.038 |  |  |
|  | Weighted mode | 15 | 0.892 ( 0.789 - 1.009 ) | 0.090 |  |  |
| Phosphatidylcholine ( 18:1_20:3 ) levels | IVW | 15 | 1.157 ( 1.033 - 1.296 ) | 0.011 | 0.448 | 0.375 |
|  | MR Egger | 15 | 1.032 ( 0.788 - 1.351 ) | 0.824 | 0.435 |  |
|  | Weighted median | 15 | 1.126 ( 0.955 - 1.328 ) | 0.159 |  |  |
|  | Weighted mode | 15 | 1.119 ( 0.890 - 1.407 ) | 0.351 |  |  |
| Triacylglycerol ( 51:2 ) levels | IVW | 9 | 0.845 ( 0.719 - 0.993 ) | 0.041 | 0.995 | 0.849 |
|  | MR Egger | 9 | 0.869 ( 0.631 - 1.198 ) | 0.420 | 0.987 |  |
|  | Weighted median | 9 | 0.829 ( 0.672 - 1.023 ) | 0.080 |  |  |
|  | Weighted mode | 9 | 0.837 ( 0.648 - 1.082 ) | 0.211 |  |  |
| Triacylglycerol ( 53:2 ) levels | IVW | 13 | 0.826 ( 0.719 - 0.949 ) | 0.007 | 0.999 | 0.999 |
|  | MR Egger | 13 | 0.826 ( 0.450 - 1.516 ) | 0.550 | 0.998 |  |
|  | Weighted median | 13 | 0.817 ( 0.688 - 0.970 ) | 0.021 |  |  |
|  | Weighted mode | 13 | 0.833 ( 0.640 - 1.085 ) | 0.200 |  |  |
| Triacylglycerol ( 53:3 ) levels | IVW | 18 | 0.889 ( 0.793 - 0.998 ) | 0.046 | 0.962 | 0.637 |
|  | MR Egger | 18 | 0.961 ( 0.686 - 1.346 ) | 0.821 | 0.950 |  |
|  | Weighted median | 18 | 0.878 ( 0.754 - 1.022 ) | 0.094 |  |  |
|  | Weighted mode | 18 | 0.880 ( 0.694 - 1.116 ) | 0.307 |  |  |

**Supplementary Table 5. MR analysis results of plasma lipid levels and colorectal cancer risk (ebi-a-GCST90013862)**
