# Supplementary material for: The main mediating lipid species in cholesterol-induced colorectal cancer risk
Source: Front Nutr. 2025 May 16;12:1453523. doi: 10.3389/fnut.2025.1453523 (PMC12124121; doi:10.3389/fnut.2025.1453523)
Supplement: Supplementary file 2 [file Image_1.pdf]

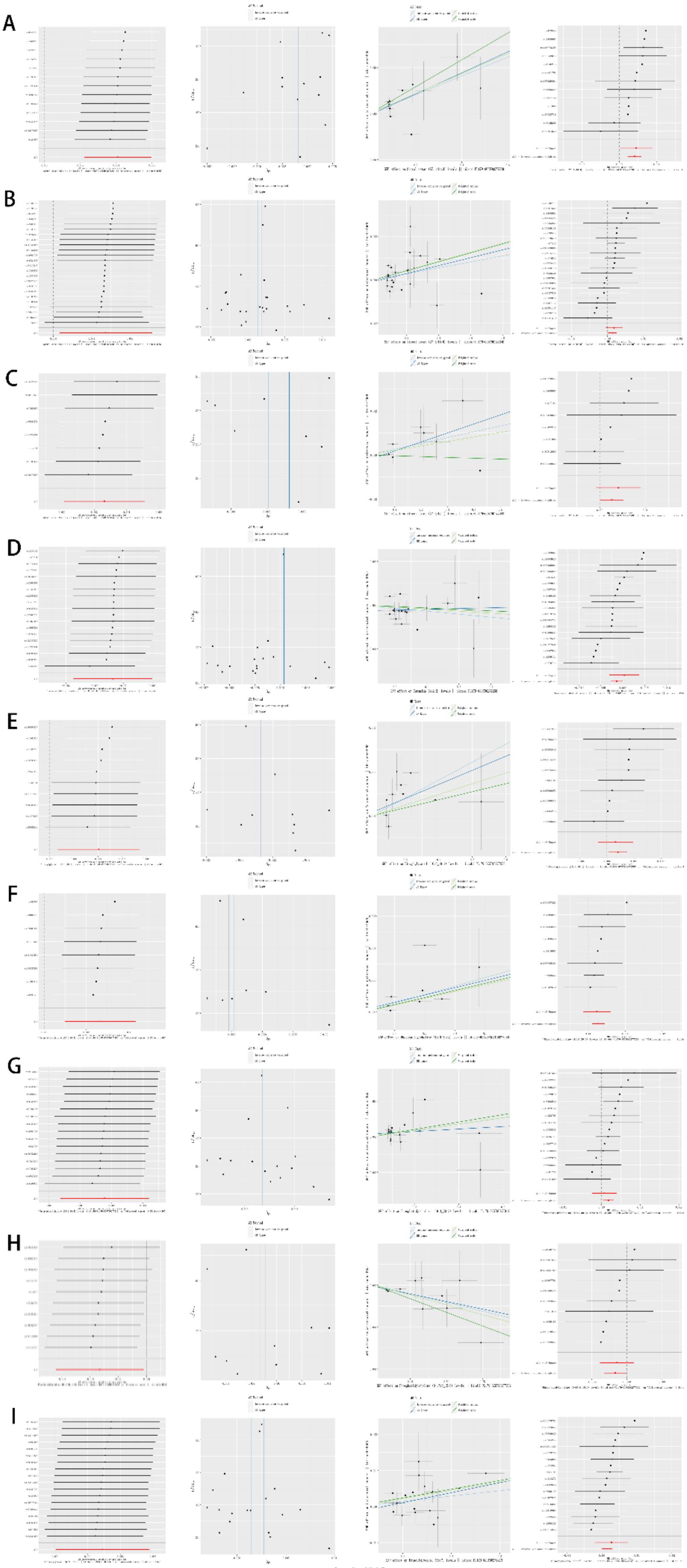

ieu-b-4965

Supplementary Fig 1. Leave-one-out sensitivity analysis of the effect of plasma lipid species on CRC and funnel plots, scatter plots and forest plots for primary MR analysis. (A. Sterol ester (27:1/14:0) levels on CRC. B. Sterol ester (27:1/16:0) levels on CRC. C. Sterol ester (27:1/17:1) levels on CRC. D. Ceramide (d42:2) levels on CRC. E. Diacylglycerol (16:0_18:1) levels on CRC. F. Phosphatidylcholine (16:0_20:3) levels on CRC. G. Phosphatidylcholine (18:1_20:3) levels on CRC. H. Phosphatidylcholine (O-17:0_15:0) levels on CRC. I. Triacylglycerol (56:7) levels on CRC.)

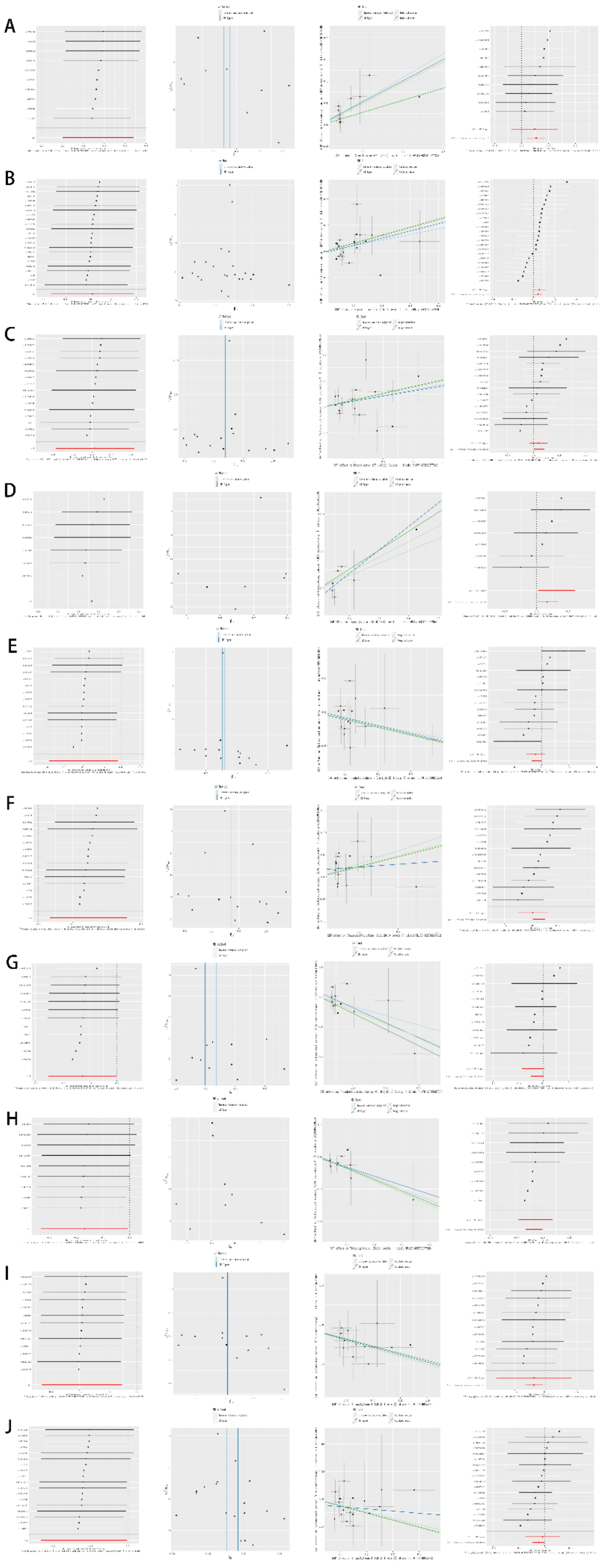

ebi-a-GCST90013866

Supplementary Fig 2. Leave-one-out sensitivity analysis of the effect of plasma lipid species on CRC and funnel plots, scatter plots and forest plots for primary MR analysis. (A. Sterol ester (27:1/14:0) levels on CRC. B. Sterol ester (27:1/16:0) levels on CRC. C. Sterol ester (27:1/18:2) levels on CRC. D. Diacylglycerol (16:0_18:1) levels on CRC. E. Phosphatidylcholine (16:1_18:2) levels on CRC. F. Phosphatidylcholine (18:1_20:3) levels on CRC. G. Phosphatidylethanolamine (O-18:2_18:1) levels on CRC. H. Triacylglycerol (51:2) levels on CRC. I. Triacylglycerol (53:2) levels on CRC. J. Triacylglycerol (53:3) levels on CRC.)

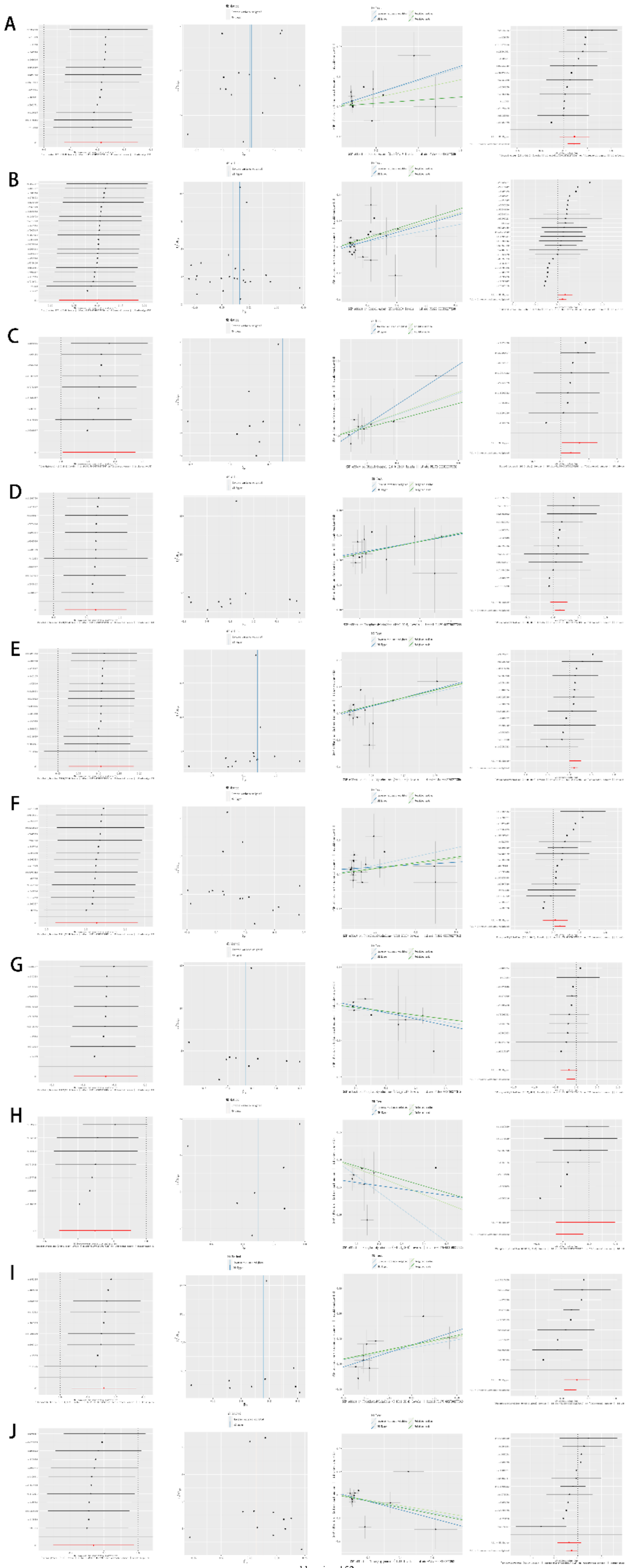

ukb-saige-153

Supplementary Fig 3. Leave-one-out sensitivity analysis of the effect of plasma lipid species on CRC and funnel plots, scatter plots and forest plots for primary MR analysis. (A. Sterol ester (27:1/14:0) levels on CRC. B. Sterol ester (27:1/16:0) levels on CRC. C. Diacylglycerol (16:0_18:1) levels on CRC. D. Phosphatidylcholine (16:0_20:5) levels on CRC. E. Phosphatidylcholine (18:0_20:5) levels on CRC. F. Phosphatidylcholine (18:1_20:3) levels on CRC. G. Phosphatidylcholine (18:2_20:1) levels on CRC. H. Phosphatidylcholine (O-16:1_18:0) levels on CRC. I. Phosphatidylcholine (O-18:1_20:4) levels on CRC. J. Triacylglycerol (51:1) levels on CRC.)

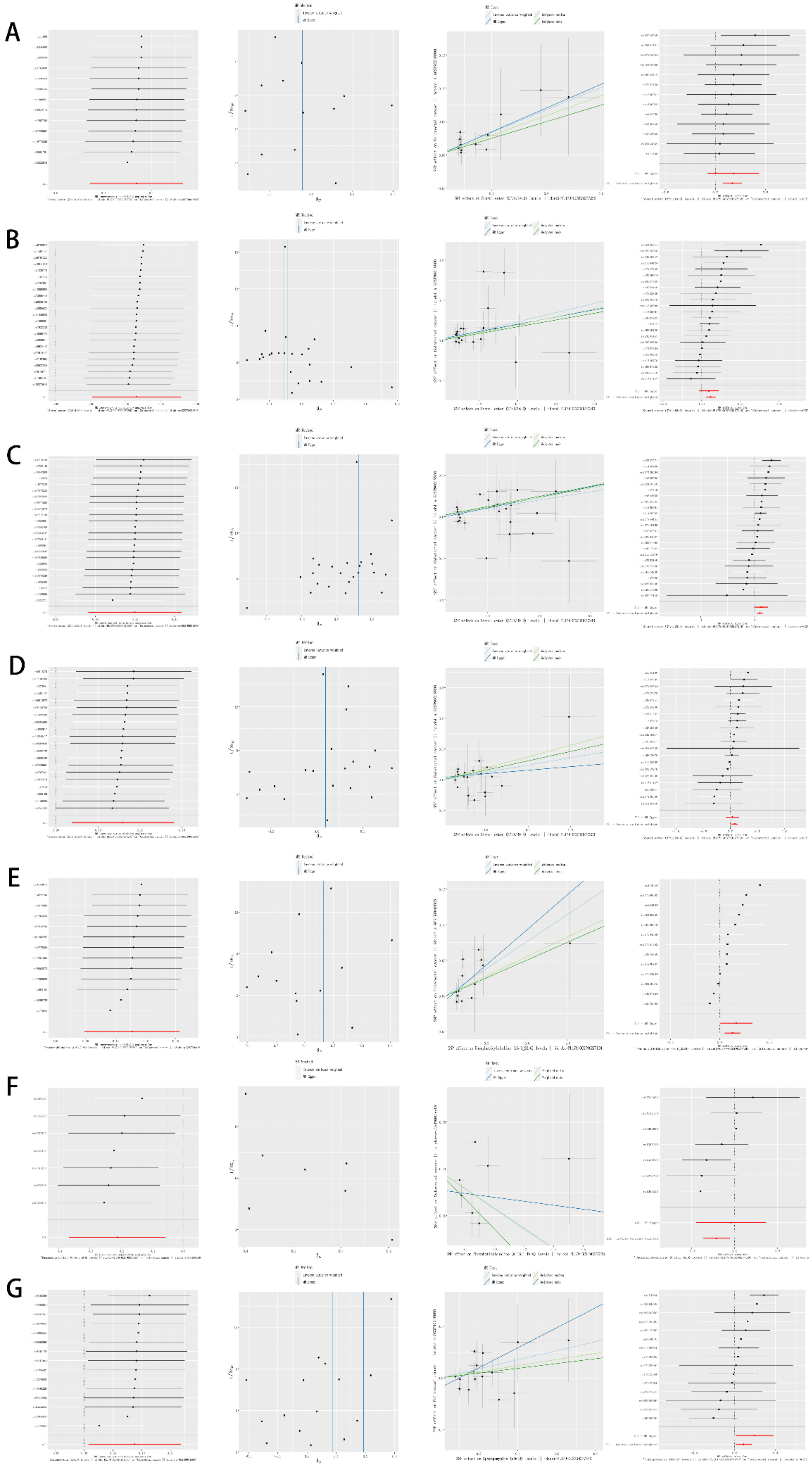

ebi-a-GCST90018808

Supplementary Fig 4. Leave-one-out sensitivity analysis of the effect of plasma lipid species on CRC and funnel plots, scatter plots and forest plots for primary MR analysis. (A. Sterol ester (27:1/14:0) levels on CRC. B. Sterol ester (27:1/16:0) levels on CRC. C. Sterol ester (27:1/20:3) levels on CRC. D. Sterol ester (27:1/20:4) levels on CRC. E. Phosphatidylcholine (16:0_22:6) levels on CRC. F. Phosphatidylcholine (O-16:1_18:0) levels on CRC. G. Sphingomyelin (d36:2) levels on CRC.)
